# Unlikely SARS-CoV-2 Transmission During Vaginal Delivery

**DOI:** 10.1007/s43032-021-00681-5

**Published:** 2021-07-22

**Authors:** Claudio Fenizia, Irma Saulle, Maria Di Giminiani, Claudia Vanetti, Daria Trabattoni, Francesca Parisi, Mara Biasin, Valeria Savasi

**Affiliations:** 1 Department of Biomedical and Clinical Sciences, University of Milan, 20157 Milan, Italy; 2 Department of Pathophysiology and Transplantation, University of Milan, 20122 Milan, Italy; 3 Unit of Obstetrics and Gynecology, ASST Fatebenefratelli-Sacco, Department of Biological and Clinical Sciences L. Sacco, University of Milan, 20157 Milan, Italy

**Keywords:** SARS-CoV-2, Pregnant women, Vaginal fluid, Rectal swabs, Intrapartum vertical transmission

## Abstract

Pregnant women display a higher risk of progression to disease and higher viral loads during infections due to their more permissive, tolerogenic immune system. However, only few studies have focused on SARS-CoV-2 intrapartum vertical transmission via vaginal secretions or faeces. The aim of this study was to investigate the presence of the virus in vaginal, rectal and blood specimens from pregnant women characterized by different COVID-19 disease severity. We enrolled 56 SARS-CoV-2-positive pregnant women, of which 46 (82%) were in the third trimester of pregnancy, 6 (10%) in the second and 4 (7%) in the first. QPCR was performed to detect the virus in vaginal and rectal swabs and in plasma samples. SARS-CoV-2 was detected in 27% of rectal swabs of pregnant women in the third trimester, while no virus particles were detected in vaginal swabs of the same patients. Furthermore, only 4% plasma samples tested positive to SARS-CoV-2. No virus was detected in newborn’s nasopharyngeal swabs. Despite the low number of subjects enrolled, our data suggest that, while theoretically possible, intrapartum vaginal or orofecal SARS-CoV-2 transmission seems to be unlikely.

## Introduction

During the last year, coronavirus disease 2019 (COVID-19) has become a global issue, proving to be a challenge for the health system of several countries worldwide. SARS-CoV-2, the causative agent of COVID-19, mostly spread via respiratory droplets from human-to-human, direct contacts or fomites, causing a wide range of clinical outcomes [1]. Despite the high number of infected individuals worldwide, only few studies have focused on intrapartum vertical transmission via vaginal secretions and faeces, and little is known about other potential routes of transmission.

The aim of this study is to investigate the presence of SARS-CoV-2 in blood, vaginal and rectal mucosa of pregnant women, to define whether the SARS-CoV-2 presence in such specimens has any correlation with the risk of SARS-CoV-2 intrapartum vertical transmission.

## Methods

This is a prospective study that includes 56 pregnant women admitted to the Department of Obstetrics and Gynaecology of “L. Sacco” University Hospital, Milan (Italy), between March 7th and April 30th, 2020. All women were enrolled upon SARS-CoV-2 detection, assessed by real-time PCR on nasopharyngeal swab. Illness categories were defined based on NIH guidelines [1]. The protocol was approved by the local Medical Ethical and Institutional Review Board (Milan, area 1, #154082020). We obtained informed written consent from the mothers to perform the procedure and analysis, according to CARE guidelines and in compliance with the Declaration of Helsinki principles.

Our pregnant population mostly included women in the third trimester close to delivery (82%), 70% of which had a vaginal delivery (VB) and 30% a caesarean section (CS). In addition, we included 6 (10%) pregnant women in the second and 4 (7%) in the first trimester. All newborns were appropriate for gestational age and healthy; for each of them, two nasopharyngeal swabs were performed right after birth and after 5 days. In addition, all newborns underwent complete medical evaluation and pharyngeal swab also at day 14 after birth, and all resulted negative to SARS-CoV-2 and healthy, as confirmed during the successive follow-up examinations.

Blood samples and vaginal and rectal swabs were collected at admission, carefully avoiding contaminations. No therapy was administered at the time of sample collection. Blood was collected in EDTA, readily transferred to the laboratory for analysis and immediately processed for plasma separation. RNA was isolated from all the specimens and tested by real-time PCR for SARS-CoV-2 as previously described [2].

## Results

Overall, the majority of the enrolled subjects in the third trimester of pregnancy displayed none-to-moderate symptoms (89%—Table 1). They all were close to delivery and indeed gave birth soon thereafter. Blood samples were obtained from 41 of these subjects. SARS-CoV-2 was detected by PCR on plasma in 2 cases (5%). One patient was at 38 weeks of gestation and had a critical illness with respiratory failure and necessity for urgent CS and invasive ventilation. The other patient was at 37 weeks of gestation, and her clinical conditions became worse after vaginal delivery. She needed oxygen support and sub-intensive care. Vaginal swabs were collected for 45 subjects in the third trimester of pregnancy, and no virus was detected. In parallel, rectal swabs were collected for 34 patients. Among these, 9 tested positive for SARS-CoV-2 (27%), including the second above-mentioned patient with viremia. Forty-five percent of women with a positive rectal swab had gastroenteric symptoms during hospitalization (diarrhoea or nausea/vomit).

**Table 1:** Maternal and pregnancy outcomes in the study population

	III Trimester *n* 46
Age, median (range)	32 (22–42)
Mode of delivery	
-VB, *n* (%) -CS, *n* (%)	32 (70) 14 (30)
Severity	
-Asymptomatic, *n* (%) -Mild, *n* (%) -Moderate, *n* (%) -Severe/critical, *n* (%)	18 (39) 11 (24) 12 (26) 5 (11)
Therapy	
-Antiviral, *n* (%) -Antibiotics, *n* (%) -Hydroxychloroquine, *n* (%) -Oxygen supply, *n* (%) -IMV, *n* (%)	12 (26) 12 (26) 11 (24) 6 (13) 1 (2)
Viremia	
-Total, *n* -Positive, *n* (%)	41 2 (5)
Vaginal swab	
-Total, *n* -Positive, *n* (%)	45 0 (0)
Rectal swab	
-Total, *n* -Positive, *n* (%)	34 9 (27)

*VB* vaginal birth, *CS* cesarean section, *IMV* invasive mechanical ventilation

No SARS-CoV-2 was detected in any of the samples collected from the subjects in the first or second trimester (data not shown). All newborns’ nasopharyngeal swabs tested negative, and they all were healthy (data not shown).

## Discussion

In the present study, all the 45 vaginal swabs collected at the third trimester of pregnancy resulted negative for viral detection, even in women with severe or critical illness (11%) or positive viremia (5%). Moreover, all infants’ nasopharyngeal swabs tested negative for SARS-CoV-2. Thus, our data suggest that the vaginal mucosa is not involved in virus spreading or replication. As during vaginal delivery, newborns can easily be contaminated by maternal faeces, which are known to often contain SARS-CoV-2 [3]; we also tested 34 rectal swabs. We detected SARS-CoV-2 in 27% of samples; nevertheless, all newborns tested negative upon delivery and at a later time. Therefore, we could not assess any case of transmission to the newborns through faecal contamination.

While other independent studies reported in utero viral transmission [4, 5], only few of them investigated the presence of SARS-CoV-2 in vaginal secretions. The majority of the reported cases were tested during menopause, which is hardly relatable with the physiology of such mucosa during pregnancy or the fertile period. However, all resulted negative for virus RNA detection [6–9]. Recently, we reported two cases (6% of the study population) of plausible in utero vertical transmission demonstrated by positive newborns’ nasopharyngeal swabs associated with detection of specific IgM antibodies or viral RNA in umbilical cord plasma [2]. The vaginal mucosa tested positive for SARS-CoV-2 in one of these two cases [2]. However, intrapartum vertical transmission could not be assessed, since the vertical transmission occurred in utero [2].

Although the number of subjects analysed in this study is not wide enough to draw firm conclusions, our data suggest that intrapartum transmission via vaginal secretions or faecal contamination seems to be unlikely.

## References

[1] Information on COVID-19 treatment, prevention and research. COVID-19 treatment guidelines. https://www.covid19treatmentguidelines.nih.gov/.

[2] Fenizia C, Biasin M, Cetin I, Vergani P, Mileto D, Spinillo A, Gismondo MR, Perotti F, Callegari C, Mancon A, Cammarata S, Beretta I, Nebuloni M, Trabattoni D, Clerici M, Savasi V (2020). Analysis of SARS-CoV-2 vertical transmission during pregnancy. Nat Commun.

[3] Rokkas T (2020). Gastrointestinal involvement in COVID-19: a systematic review and meta-analysis. Ann Gastroenterol.

[4] Zeng H, Xu C, Fan J, Tang Y, Deng Q, Zhang W, Long X (2020). Antibodies in infants born to mothers with COVID-19 pneumonia. JAMA.

[5] Dong L, Tian J, He S, Zhu C, Wang J, Liu C, Yang J (2020). Possible vertical transmission of SARS-CoV-2 from an infected mother to her newborn. JAMA.

[6] Qiu L, Liu X, Xiao M, Xie J, Cao W, Liu Z, Morse A, Xie Y, Li T, Zhu L (2020). SARS-CoV-2 is not detectable in the vaginal fluid of women with severe COVID-19 infection. Clin Infect Dis.

[7] Cui P, Chen Z, Wang T, Dai J, Zhang J, Ding T, Jiang J, Liu J, Zhang C, Shan W, Wang S, Rong Y, Chang J, Miao X, Ma X, Wang S (2020). Severe acute respiratory syndrome coronavirus 2 detection in the female lower genital tract. Am J Obstet Gynecol.

[8] Peng Z, Wang J, Mo Y, Duan W, Xiang G, Yi M, Bao L, Shi Y (2020). Unlikely SARS-CoV-2 vertical transmission from mother to child: A case report. J Infect Public Health.

[9] Baud D, Greub G, Favre G, Gengler C, Jaton K, Dubruc E, Pomar L (2020). Second-trimester miscarriage in a pregnant woman with SARS-CoV-2 infection. JAMA.

